# Impact of aerobic exercises on selected inflammatory markers and immune system response among patients with sickle cell anemia in asymptomatic steady state

**DOI:** 10.4314/ahs.v18i1.15

**Published:** 2018-03

**Authors:** Shehab M Abd El-Kader, Fadwa M Al-Shreef

**Affiliations:** 1 Department of Physical Therapy, Faculty of Applied Medical Sciences, King Abdulaziz University, Jeddah, Saudi Arabia; 2 Department of Medical Laboratory Technology, Faculty of Applied Medical Sciences, King Abdulaziz University, Jeddah, Saudi Arabia

**Keywords:** Cytokines, immune parameters, aerobic exercise, sickle cell disease, stable state

## Abstract

**Background & objective:**

Sickle cell anemia (SCA) is well recognized as a chronic inflammatory disease. Despite progress in therapy, SCA remains a cause of significant morbidity and mortality. The relationship between exercise and immune function has been of great interest to the scientific community and the lay public. The aim of this study was to measure the impact of aerobic exercise training on the immunologic parameters and inflammatory cytokines of patients with sickle cell anemia (SCA) in asymptomatic steady state.

**Material and methods:**

Sixty asymptomatic sickle cell anemia patients were involved in this study, their age ranged from 25– 40 years and were assigned to two sub-groups; group (A) received aerobic exercise training for 12 weeks, however group (B) received no training intervention for 12 weeks. Parameters of CD3, CD4 and CD8 were quantified, Leukocyte, differential counts, IL-6 and TNF-α were measured before and after 12 weeks, at the end of the study.

**Results:**

The mean values of CD3, CD4 and CD8, leukocyte, monocytes counts, IL-6 and TNF-α were significantly decreased in group (A), while group (B) showed non-significant changes in these parameters. Also; there were significant differences between mean levels of the investigated parameters in group (A) and group (B) after treatment.

**Conclusion:**

The current study provides evidence that aerobic exercise training improves inflammatory markers and immune system in patients with sickle cell anemia (SCA) in asymptomatic steady state.

## Introduction

Sickle cell anemia (SCA) is now the world's most common genetic defect. It is estimated that 5% of the population carries a haemoglobinopathy trait worldwide, there are an estimated 300,000 babies born annually worldwide with a severe hemoglobin disorder^1^. Despite the significant increase in research and number of published articles, many aspects of the pathophysiology of the disease and its complications remain elusive^2^.

Sickle cell anemia (SCA) is a chronic condition presenting in people homozygous for hemoglobin S (HbS)^3^. Tissue ischemia due to vascular occlusion causing infarctive tissue damage, which in turn initiates secondary inflammatory responses^4^,^5^.

People with SCA are also at a higher risk of stroke, acute chest syndrome, leg ulcers, pulmonary hypertension, and other complications^6^. Although SCA generally is a benign and asymptomatic state, red blood cell sickling and splenic infarction can occur at low oxygen levels such as at high altitudes^7^. Causes of death among patients with SCA include cardiopulmonary causes such as cardiac arrest, heart failure, and pulmonary embolism, infections, stroke, and multi-organ failure^8^. Life expectancy in HbS from a multicenter study in the USA in 1994 was estimated at 42 for men and 48 for women^9^. African children with SCA mortality is still high as an early-life mortality of 50%–90% among children born in Africa with SCA^10^.

Sickle cell disease has a deleterious effect on immune system functions, and thus children with SCA are at increased risk of life-threatening infections, especially with *Streptococcus pneumoniae* and *Haemophilus influenzae*^11^. Moreover, infection has long been recognized as one of the most common precipitants of crisis in SCA^12^.

As there is limitation in studies reporting the benefits of aerobic exercises on immune system response among patients with sickle cell anemia (SCA). This study aimed to examine effects of aerobic exercise training on selected inflammatory markers and immune parameters among patients with sickle cell anemia (SCA) in asymptomatic steady state.

## Subjects and methods

### Subjects

Sixty sickle cell anemia Saudi patients in stable state that presented at the Haematology Department, King Abdalaziz University Hospital, were recruited into the study. Cases were selected from patients whose blood samples were submitted to the hematology section for hemoglobin electrophoresis, which was either advised by their treating doctor or was performed to confirm a positive sickling test. All confirmed patients of sickle cell haemoglobinopathy diagnosed by presence of Hemoglobin ‘S’ band on hemoglobin electrophoresis and only homozygous Sickle cell disease patients (patients whose electrophoresis showed presence of Haemoglobin ‘S’ band with or without Haemoglobin ‘F’ band — HbSS genotype) were included in the study. Each patient was assessed clinically to confirm steady state. The steady state was defined as the period free of crisis extending from at least three weeks since the last clinical event and three months or more since the last blood transfusion, to at least one week before the start of a new clinical event. Some demographic data (age, gender, weight and height) of both the subjects and controls were also documented.

**Figure (1):**
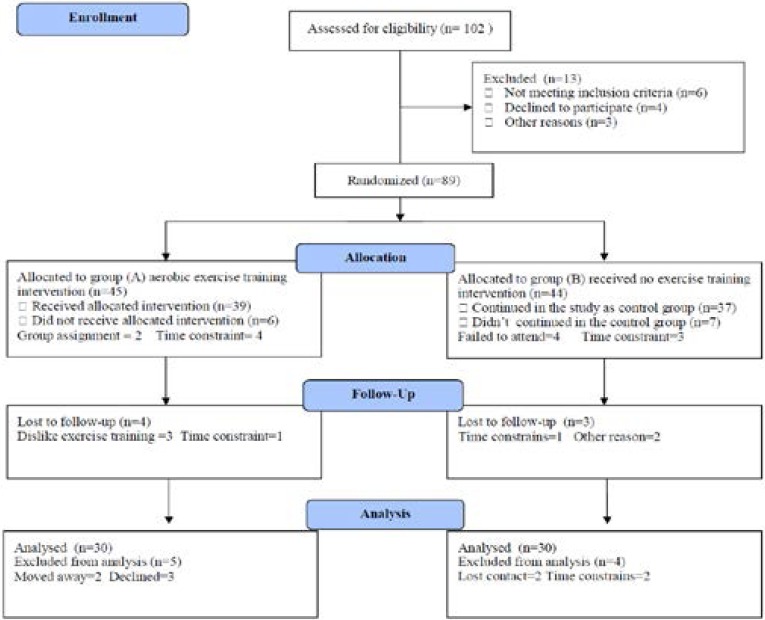
Subjects screening and recruitment CONSORT diagram.

Exclusion criteria included sickle cell disease patient with concurrent HIV or overt infection. Also, SCA patient with painful vaso-occlusive crisis (VOC) with bone or joint pain, or multiple sites of pain, necessitating hospital admission and analgesic administration. Participants were divided into two groups; group (A) received treadmill aerobic exercise training on treadmill. However, group (B) received no exercise training. The CONSORT diagram outlining the details of the screening, run-in and randomization phases of the study and reasons for participant exclusion can be found in figure (1). All participants signed the informed consent.

## Methods

### Measurements

The following measurements were taken before the study and after 3 months, at the end of the study.

#### A Inflammatory cytokines

Blood samples were drained from the antecubital vein after a 12-h fasting, the blood samples were centrifuged at + 4 °C (1000 = g for 10 min). Interleukin-6 (IL-6) level was analyzed by “Immulite 2000” immunassay analyzer (Siemens Healthcare Diagnostics, Deerfield, USA). However, tumor necrosis factor-alpha (TNF-α) was measured by ELISA kits (ELX 50) in addition to ELISA microplate reader (ELX 808; BioTek Instruments, USA). All analyses were done by Hitachi 7170 Autoanalyser (Tokyo, Japan) and kits (Randox).

#### B Analysis of peripheral blood cells

The analysis of peripheral blood cells (e.g., total and differential count) was performed on a Beckman Coulter AcT 5diff hematology analyzer. The values were expressed in percentages and absolute numbers.

#### C Flow cytometry analysis

The human leukocyte differentiation antigens CD3, CD4 and CD8 (Beckman Coulter, Marseille, France) Five microliters of appropriate monoclonal antibody was added to 50 µL of a whole-blood sample and incubated for 15 minutes at room temperature. Thereafter, the erythrocytes were lysed with 125 µL of a lysing solution, OptiLyse C, for 10 minutes. The reaction was stopped by the addition of 250 µL phosphate-buffered saline. The samples were analyzed by flow cytometry using Cytomics FC 500 and CXP software (Beckman Coulter).The leukocyte subsets were defined by forward- and side-scatter pattern. The negative control value was determined by a fluorescence background and antibody-nonspecific staining.

### Procedures

Following the previous evaluation, all patients enrolled randomly into the following groups:

1. The training group (Group A) received aerobic exercise training for 12-weeks on the treadmill (Enraf Nonium, Model display panel Standard, NR 1475.801, Holand) which was conducted according to recommendation of aerobic exercise application approved by the American College of Sports Medicine^13^. Training program included 5 minutes for warming -up in the form of range motion and stretching exercises, 30 minutes of aerobic exercise training with intensity equal 60–70% of the individual maximum heart rate followed by cooling down for 10 minutes (on treadmill with low speed and without inclination). Participants had 3 sessions /week for 3 months with close supervision of a physical therapist.
2. The control group (Group B) received no exercise intervention.

### Statistical analysis

The mean values of the investigated parameters obtained before and after three months in both groups were compared using paired “t” test. Independent “t” test was used for the comparison between the two groups (P<0.05).

## Results

The two groups were considered homogeneous regarding the demographic variables (table 1). The mean age of group (A) was 25.16±7.14 years, and the mean age of group (B) was 24.54±7.63 years. There were no significant differences in weight, height, body mass index (BMI), systolic blood pressure and diastolic blood pressure between both groups.

**Table 1:** Comparison of demographic variables between both groups.

	Group (A)	Group (B)	Significance
**Age** (year)	26.54 ± 6.73	24.91 ± 7.32	P>0.05
**Weight** (kg)	38.76 ± 8.65	40.13 ± 10.11	P>0.05
**Height** (cm)	167.42 ± 10.21	166.85 ± 9.37	P>0.05
**BMI** (kg/m^2^)	18.56 ± 3.18	19.74 ± 3.25	P>0.05
**SBP** (mmHg)	116.71 ± 10.24	118.27 ± 12.57	P>0.05
**DBP** (mmHg)	75.83 ± 6.22	78.61 ± 5.73	P>0.05

BMI: Body Mass Index; SBP: Systolic blood pressure; DBP: Diastolic blood pressure.

There was a 28.1%, 24.7%, 33.1%, 32.8%, 36.2%, 35.3%, 20.3% and 37.2% reduction in mean values of tumor necrosis factor-α (TNF-α), interleukin-6 (IL-6), white blood cells count, total neutrophil count, Monocytes , CD3 count , CD4 count and CD8 count respectively in the training group (table 2).

**Table 2:** Mean value and significance of TNF-α, IL-6, white blood cells count, total neutrophil count, monocytes, CD3 count, CD4 count and CD8 count in group (A) before and at the end of the study.

	Mean + SD		t-value	Significance
	Pre	Post		
**TNF-α** (pg/mL)	4.51 ± 1.63*	3.24 ± 1.35	6.88	P <0.05
**IL-6** (pg/mL)	2.34 ± 0.92*	1.76 ± 0.85	5.76	P <0.05
**WBC count** (10^9^/µL)	9.17 ± 3.87*	6.13 ± 2.92	7.14	P <0.05
**Total neutrophil**count (10^9^/µL)	6.33 ± 2.62*	4.25 ± 2.31	6.28	P <0.05
**Monocytes** (10^9^/µL)	0.58 ±0.14*	0.37 ±0.11	5.24	P <0.05
**CD3 count** (10^9^/L)	1.95 ± 0.87*	1.26 ± 0.72	4.23	P<0.05
**CD4 count** (10^9^/L)	1.52 ± 0.75*	1.21 ± 0.61	4.35	P <0.05
**CD8 count** (10^9^/L)	0.86 ± 0.27*	0.54 ± 0.13	4.12	P <0.05

TNF-α: tumor necrosis factor — alpha; IL-6: Interleukin-6; WBC: white blood cells;

(*) indicates a significant difference between the two groups, P < 0.05.

While, there was a 3.4%, 3.7%, 2.5%, 2.1%, 3.7%, 6.5%, 6.3% and 5.4% increase in mean values of the same variables in the control group. The mean values of TNF-α, IL-6, white blood cells count, total neutrophil count, monocytes, CD3 count, CD4 count and CD8 count decreased significantly in the training group, however the results of the control group were not significant (table 3). Also, there were significant differences between both groups at the end of the study (table 4).

**Table 3:** Mean value and significance of TNF-α, IL-6, white blood cells count, total neutrophil count, monocytes, CD3 count, CD4 count and CD8 count in group (B) before and after the study.

	Mean + SD		t-value	Significance
	Pre	Post		
**TNF-α** (pg/mL)	4.38 ± 1.51	4.53 ± 1.55	1.24	P>0.05
**IL-6** (pg/mL)	2.16 ± 0.83	2.24 ± 0.85	1.16	P>0.05
**WBC** **count** (10^9^/µL)	9.23 ± 3.57	9.46 ± 3.62	1.34	P>0.05
**Total** **neutrophil** count (10^9^/µL)	6.18 ± 2.45	6.31 ± 2.48	1.15	P>0.05
**Monocytes** (10^9^/µL)	0.54 ± 0.16	0.56 ± 0.17	0.98	P>0.05
**CD3 count** (10^9^/L)	1.83 ± 0.91	1.95 ± 0.98	0.85	P>0.05
**CD4 count** (10^9^/L)	1.41 ± 0.85	1.50 ± 0.93	0.72	P>0.05
**CD8 count** (10^9^/L)	0.73 ± 0.26	0.77 ± 0.34	0.68	P>0.05

TNF-α: tumor necrosis factor — alpha; IL-6: Interleukin-6; WBC: white blood cells;

(*) indicates a significant difference between the two groups, P < 0.05.

**Table 4:** Mean value and significance of TNF-α, IL-6, white blood cells count, total neutrophil count, monocytes, CD3 count, CD4 count and CD8 count in group (A) and group (B) at the end of the study.

	Mean + SD		t-value	Significance
	Group (A)	Group (B)		
**TNF**-α (pg/mL)	3.24 ± 1.35*	4.53 ± 1.55	6.17	P <0.05
**IL-6** (pg/mL)	1.76 ± 0.85*	2.24 ± 0.85	5.38	P <0.05
**WBC** count (10^9^/µL)	6.13 ± 2.92*	9.46 ± 3.62	6.27	P <0.05
**Total** **neutrophil** count (10^9^/µL)	4.25 ± 2.31*	6.31 ± 2.48	5.88	P <0.05
**Monocytes** (10^9^/µL)	0.37 ±0.11*	0.56 ± 0.17	4.36	P <0.05
**CD3 count** (10^9^/L)	1.26 ± 0.72*	1.95 ± 0.98	3.91	P <0.05
**CD4 count** (10^9^/L)	1.21 ± 0.61*	1.50 ± 0.93	3.65	P <0.05
**CD8 count** (10^9^/L)	0.54 ± 0.13*	0.77 ± 0.34	3.76	P <0.05

TNF-α: tumor necrosis factor — alpha; IL-6: Interleukin-6; WBC: white blood cells;

(*) indicates a significant difference between the two groups, P < 0.05.

## Discussion

Sickle cell disease is well recognized as a chronic inflammatory disease^14^ as cytokines are elevated in steady state^15^. It is widely accepted that physical exercise may bring about changes in the immune system. Despite the high interpersonal variation, it is widely accepted that physical exercise may promote changes in the immune system^21^,^22^.

To our knowledge, this is the first study addressing anthropometric, inflammatory and immunological parameters of patients with SCA after 12 weeks of concurrent training. We observed increased markers of immune system and reduction of markers of systemic inflammation. We also observed lower levels of CD3, CD4 and CD8, Leukocyte, differential counts, IL-6 and TNF-α after 12 weeks of concurrent aerobic training. The results of our study agreed with several previous studies suggesting that aerobic exercise promotes the modulation of immune system systemic inflammation.

Kohut et al. randomly assigned a group of elderly to either an aerobic exercise treatment or a flexibility/strength exercise treatment 3 days/week, 45 min/day for 10 months. Aerobic exercise treatment group experienced significant reductions in serum CRP and IL-6 compared to flexibility/strength exercise treatment group, whereas TNF-α declined in both groups^23^. Also, Shih and colleagues reported significant reductions in all anthropometric, metabolic, interleukin-6 and C-reactive protein after completion of 12-week exercise program^24^. Similarly, Goldhammer and colleagues found significant reduction in CRP and IL-6 among twenty-eight patients with coronary heart disease following a 12-week aerobic exercise training program^26^. In addition, Mattusch et al. found 9 months of marathon training (n=12) to reduce CRP levels by 31%, with no change in the non-training control group (n=10)^29^. While, Hayashino and colleagues checked fourteen randomized controlled trials (824 patients with type 2 diabetes) were included in their meta-analysis. Exercise was associated with a significant in CRP and IL-6^25^. The three possible mechanisms of exercise anti-inflammatory effects include reduction in visceral fat mass^34^; reduction in the circulating numbers of pro-inflammatory monocytes^35^ and an increase in the circulating numbers of regulatory T cells^36^. A major finding of this work is that aerobic exercise training may restore immune function among patients with SCA. The results of our study are compatible with several previous studies suggesting that exercise training promotes the modulation of immune system markers. Woods et al. found that 6 months of supervised aerobic exercise training (composed of 30-minutes of brisk walking 3 times/week) in the elderly increased T-cell proliferation compared to controls in previously sedentary elderly^37^. While, Kursat et al. concluded that regular and moderate aerobic exercise on treadmill for 30 minutes has favorable effects on the immune system by increasing immunoglobulines (IgA, IgG and IgM levels ) which are potent protective factors^38^. Also, Buyukyazi proved that baseline NK cell percentage, and serum IgA and IgM concentrations were significantly higher among eleven elderly male athletes performing regular aerobic exercise for about 4.4 ± 1.4 day/week for 10.0 ± 8.1 hour/week than eleven male individuals at similar ages leading a sedentary life who were taken as a control group^39^. Moreover, the possible mechanisms of exercise immune system modulating effects include decreased levels of pro-inflammatory cytokines TNF-α42,IL-6 43 and C-reactive protein44 along with an increase in the interleukin -10 (IL-10)43.

The current study has important strengths and limitations. The major strength is the supervised nature of the study. Supervising physical activity removes the need to question compliance or to rely on activity questionnaires. Further, all exercise sessions were supervised and adherence to the activities was essentially 100%. Moreover, the study was randomized; hence, we can extrapolate adherence to the general population. On the other hand, the major limitations is only patients with sickle cell anemia (SCA) in asymptomatic steady state were enrolled in the study, so the value of this study is only related to SCA in asymptomatic steady state, also a small sample size in both groups may limit the possibility of generalization of the findings in the present study. Finally, within the limit of this study, aerobic exercise training is recommended for modulation of low grade systemic inflammation and immune system among patients with SCA in asymptomatic steady state. Further researches are needed to explore the impact of aerobic exercise training on quality of life and other biochemical parameters among patients with SCA.

## Conclusion

The current study provides evidence that aerobic exercise training improves inflammatory markers and immune system in patients with sickle cell anemia (SCA) in asymptomatic steady state.
